# Supplementary material for: Dominant immune tolerance in the intestinal tract imposed by RelB-dependent migratory dendritic cells regulates protective type 2 immunity
Source: Nat Commun. 2024 Oct 23;15:9143. doi: 10.1038/s41467-024-53112-9 (PMC11500181; doi:10.1038/s41467-024-53112-9)
Supplement: Supplementary file 1 — Supplementary Information [file 41467_2024_53112_MOESM1_ESM.pdf]

Supplementary Figures 1-7

for

Dominant immune tolerance in the intestinal tract imposed by RelB-dependent migratory dendritic cells regulates protective type 2 immunity

Running title: RelB expression limits dominant immune tolerance

Anna-Lena Geiselhöringer[1], Daphne Kolland[1], Arisha Johanna Patt[1], Linda Hammann[2], Amelie Köhler[1], Luisa Kreft[1 #], Nina Wichmann[1], Miriam Hils[3], Christiane Ruedl[4], Marc Riemann[5], Tilo Biedermann[3], David Anz[2, 6], Andreas Diefenbach[7], David Voehringer[8], Carsten B. Schmidt-Weber[1, 9], Tobias Straub[10], Maria Pasztoi[1], Caspar Ohnmacht[1] *

[1] Center of Allergy and Environment (ZAUM), Technical University and Helmholtz Center Munich, Germany.
[2] Division of Clinical Pharmacology, LMU University Hospital, LMU Munich, Germany.
[3] Department of Dermatology and Allergy Biederstein, School of Medicine and Health, Technical University of Munich, Munich, Germany.
[4] School of Biological Sciences, Nanyang Technological University Singapore, Singapore.
[5] Leibniz Institute on Aging, Fritz Lipmann Institute, 07745 Jena, Germany.
[6] Department of Medicine II, LMU University Hospital, LMU Munich, Germany.
[7] Laboratory of Innate Immunity, Institute of Microbiology, Infectious Diseases and Immunology, Charité-Universitätsmedizin Berlin, 12203 Berlin, Germany; Mucosal and Developmental Immunology, Deutsches Rheuma-Forschungszentrum, an Institute of the Leibniz Association, 10117 Berlin, Germany.
[8] Department of Infection Biology, University Hospital Erlangen and Friedrich-Alexander University Erlangen-Nuremberg (FAU), Erlangen, 91054, Germany.
[9] Member of the German Center of Lung Research (DZL), Partner Site Munich, Munich, Germany.
[10] Bioinformatics Core Unit, Biomedical Center, Ludwig-Maximilians-University, 82152 Planegg, Germany.

[#] Current address: Immatics Biotechnologies GmbH, Paul-Ehrlich-Str. 15, 72076 Tuebingen, Germany.

* Corresponding author:

Caspar Ohnmacht, Center of Allergy and Environment (ZAUM), Technical University and Helmholtz Center Munich, Germany. Email: caspar.ohnmacht@helmholtz-munich.de

Supplementary figures

Supplementary figure 1

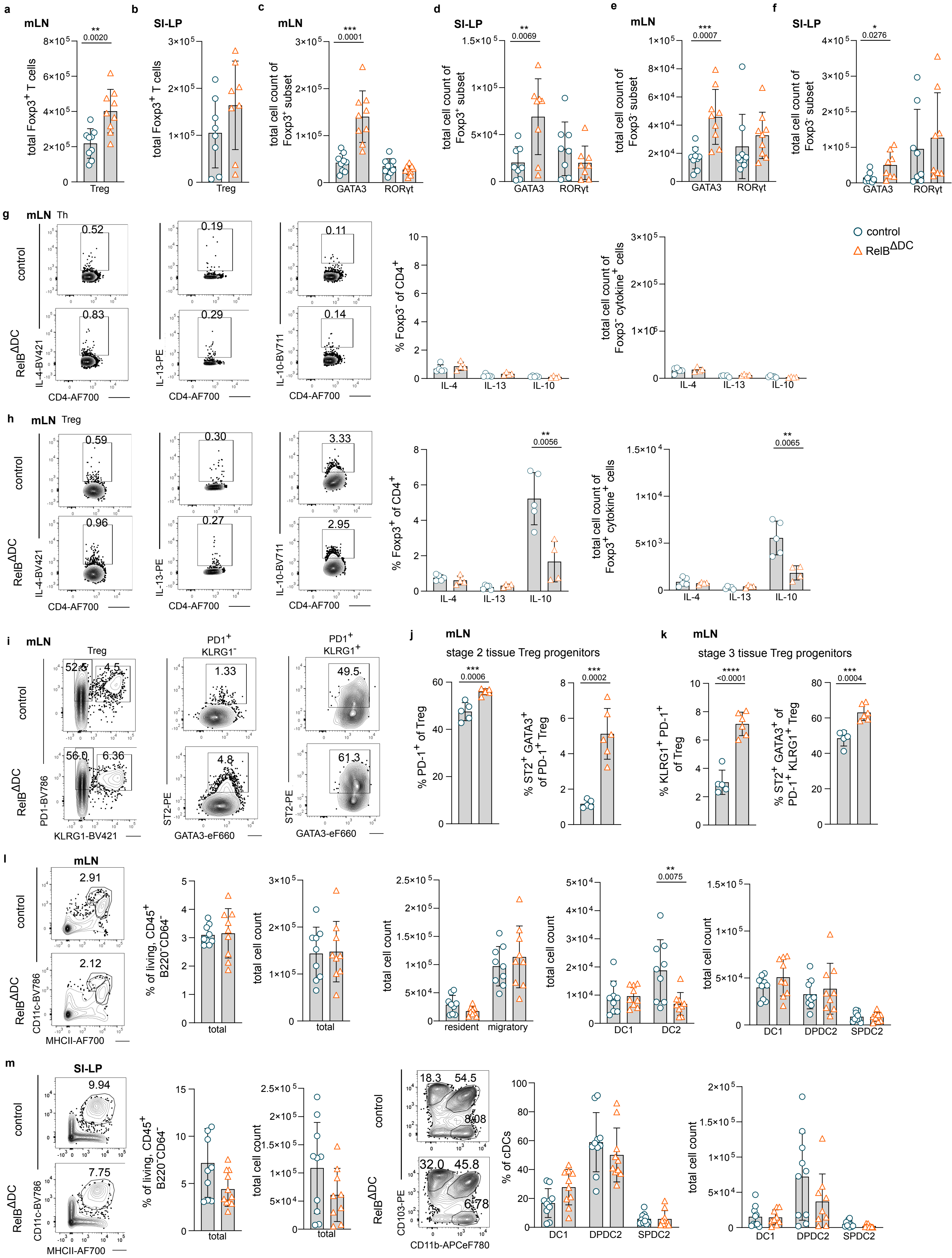

Supplementary Figure 1: **Influence of RelB ablation on total T cells and DCs at steady state**

a-h Flow cytometric analysis of T cell populations in control and $RelB^{\Delta DC}$ mice at steady state. **a** Total cell number of $Foxp3^+$ T cells in mesenteric lymph nodes (mLN, control *n* = 9, $RelB^{\Delta DC}$ *n* = 9). **b** Total cell numbers of $Foxp3^+$ T cells in lamina propria of the small intestine (SI-LP, control *n* = 8, $RelB^{\Delta DC}$ *n* = 8). **c** Total cell number of GATA3- and RORγt- expressing $Foxp3^+$ Treg cells in mLN (control *n* = 9, $RelB^{\Delta DC}$ *n* = 9). **d** Total cell numbers of GATA3- and RORγt-expressing $Foxp3^+$ Treg cells in SI-LP (control *n* = 8, $RelB^{\Delta DC}$ *n* = 8). **e** Total cell number of GATA3- and RORγt- expressing $Foxp3^-$ Th cells in mLN (control *n* = 9, $RelB^{\Delta DC}$ *n* = 9). **f** Total cell numbers of GATA3- and RORγt- expressing $Foxp3^-$ Th cells in SI-LP (control *n* = 8, $RelB^{\Delta DC}$ *n* = 8). **g**, **h** Flow cytometric analysis of cytokine levels of T cells in control and $RelB^{\Delta DC}$ mice at steady state. **g** Representative contour plots (left), quantification (middle) and total cell counts (right) of IL-4, IL-13 and IL-10 producing $Foxp3^-$Th cells in mLN (control *n* = 5, $RelB^{\Delta DC}$ *n* = 4). **h** Representative contour plots (left), quantification (middle) and total count (right) of IL-4, IL-13 and IL-10 producing $Foxp3^+$ Treg cells in mLN (control *n* = 5, $RelB^{\Delta DC}$ *n* = 4). **i-k** Flow cytometric analysis of tissue Treg progenitors in mLN of control and $RelB^{\Delta DC}$ mice at steady state. Representative flow cytometry plots (**i**) of PD-1^+ $KLRG1^-$ stage 2 tissue Treg progenitors and PD-$1^+$$KLRG1^+$ stage 3 tissue Treg progenitor frequencies among $Foxp3^+$ T cells (left), $GATA3^+$$ST2^+$ cells among stage 2 tissue Treg progenitors (middle) and $GATA3^+$$ST2^+$ cells among stage 3 tissue Treg progenitors (right). **j** Quantification of stage 2 tissue Treg progenitors (left) and $GATA3^+$$ST2^+$ cell frequencies among stage 2 tissue Treg (right) (control *n* = 5, $RelB^{\Delta DC}$ *n* = 6). **k** Quantification of stage 3 tissue Treg progenitor (left) and $GATA3^+$$ST2^+$ cell frequencies (right) among stage 3 tissue Treg cells (control *n* = 5, $RelB^{\Delta DC}$ *n* = 6). **l, m** Flow cytometric analysis of DC populations in control and $RelB^{\Delta DC}$ mice at steady state. **l** Representative flow cytometry plots (1^{st}), frequencies (2^{nd}) and total cell counts (3^{rd}) of cDCs (live/dead$CD45^+$$CD64^-$$B220^-$$CD11c^{high}$$MHCII^{high}$) in mLN. Total cell count of resident and migratory DCs (4^{th}), resident DC subsets (5^{th}) and migratory DC subsets (6^{th}) (control *n* = 9, $RelB^{\Delta DC}$ *n* = 9) **m** Representative flow cytometry plots (1^{st}), frequencies (2^{nd}) and total count (3^{rd}) of cDCs (live/dead$CD45^+$$CD64^-$$B220^-$$CD11c^{high}$$MHCII^{high}$) in SI-LP. Representative flow cytometry plots (4th), frequency (5th) and total cell count (6th) of DC subsets in SI-LP (control *n* = 10, $RelB^{\Delta DC}$ *n* = 11). Each dot represents an individual mouse and mean ± SD from two independent experiments (**a-f, i-m**) or one experiment (**g**, **h**) is shown. Statistical analysis was performed using two-tailed students t-test. P value of <0.05 was considered statistically significant with *p <0.05, **p< 0.01, ***p < 0.001, ****p <0.0001. Source data are provided as a Source Data file.

Supplementary figure 2

scRNASeq of cDCs in SI-LP

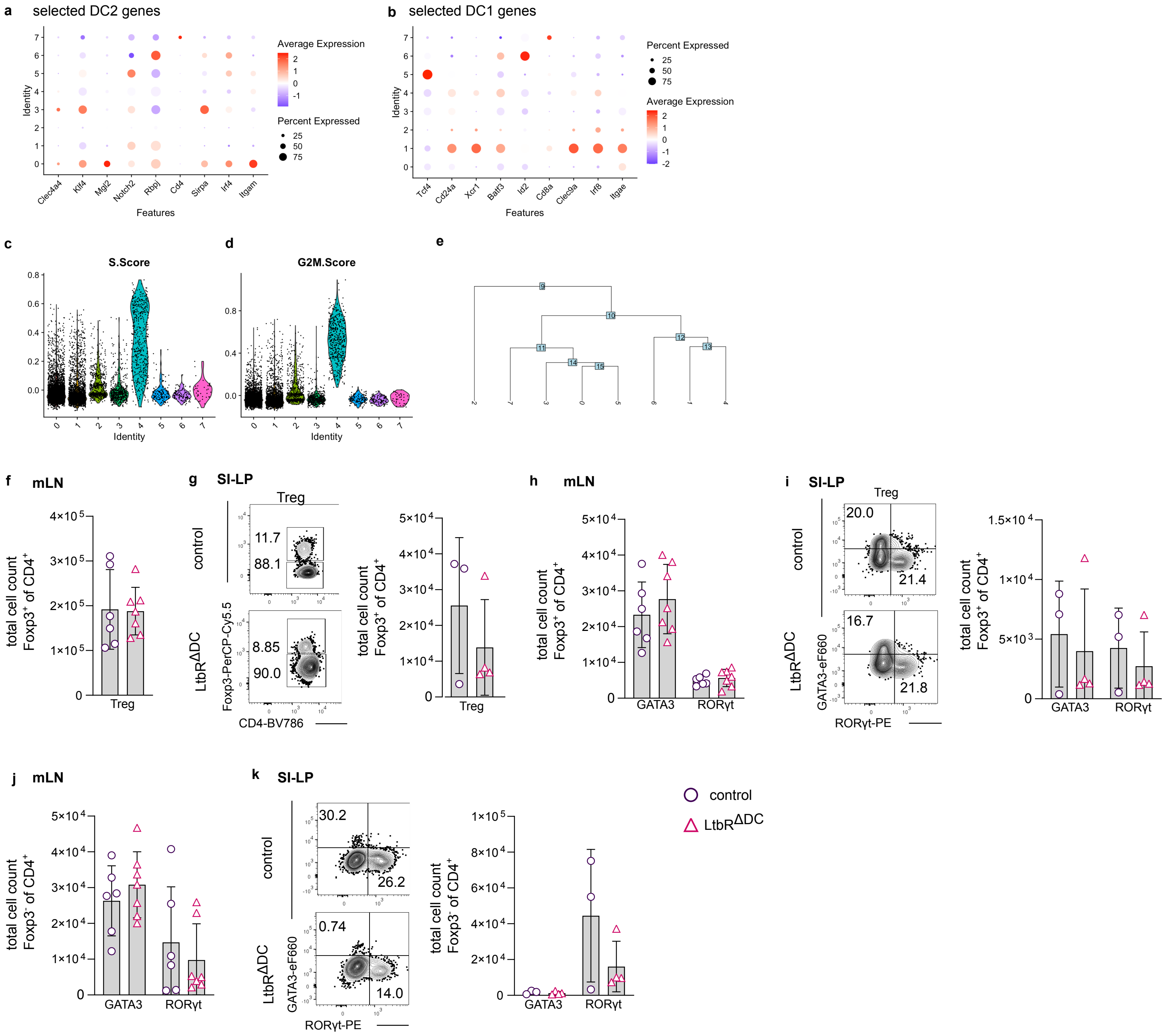

Supplementary Figure 2: Identification of DC subsets in scRNASeq clusters from SI-LP

Characterization of clusters shown in UMAP of scRNASeq of $CD11c^{+}MHCII^{+}$ cells from lamina propria of the small intestine (SI-LP) of control and $RelB^{\Delta DC}$ mice in **Fig. 2a**. **a** Feature plot depicting expression levels of DC2 specific genes in indicated clusters. **b** Feature plot depicting expression levels of DC1 specific genes in indicated clusters. **c, d** S score (**c**) and G2M score (**d**) of indicated clusters. **e** Cluster tree analysis identified clusters from **Fig. 2a**. **f-k** Flow cytometric analysis of T cell populations in control and $LtbR^{\Delta DC}$ mice at steady state. **f** Total cell number of $Foxp3^{+}$ T cells from mesenteric lymph node (mLN, control n = 6, $LtbR^{\Delta DC}$ n = 7). **g** Representative contour plots (left) and total cell numbers (right) of $Foxp3^{+}$ T cells from SI-LP (control n = 3, $LtbR^{\Delta DC}$ n = 4). **h** Total cell number of GATA3 and RORγt expressing $Foxp3^{+}$ Treg cells in mLN (control n = 6, $LtbR^{\Delta DC}$ n = 7). **i** Representative contour plots (left) and total cell numbers (right) of GATA3 and RORγt expressing $Foxp3^{+}$ Treg cells in SI-LP (control n = 3, $LtbR^{\Delta DC}$ n = 4). **j** Total cell number of GATA3 and RORγt expressing $Foxp3^{-}$ Th cells in mLN (control n = 6, $LtbR^{\Delta DC}$ n = 7). **k** Representative contour plots (left) and total cell numbers (right) of GATA3 and RORγt expressing $Foxp3^{-}$ Th cells in SI-LP (control n = 3, $LtbR^{\Delta DC}$ n = 4). Each dot represents an individual mouse and mean ± SD from two independent experiments is shown. Statistical analysis was performed using two-tailed students t-test. P value of <0.05 was considered statistically significant with *p <0.05, **p< 0.01, ***p < 0.001, ****p <0.0001. Source data are provided as a Source Data file.

Supplementary figure 3

scRNASeq of cDCs in mLN

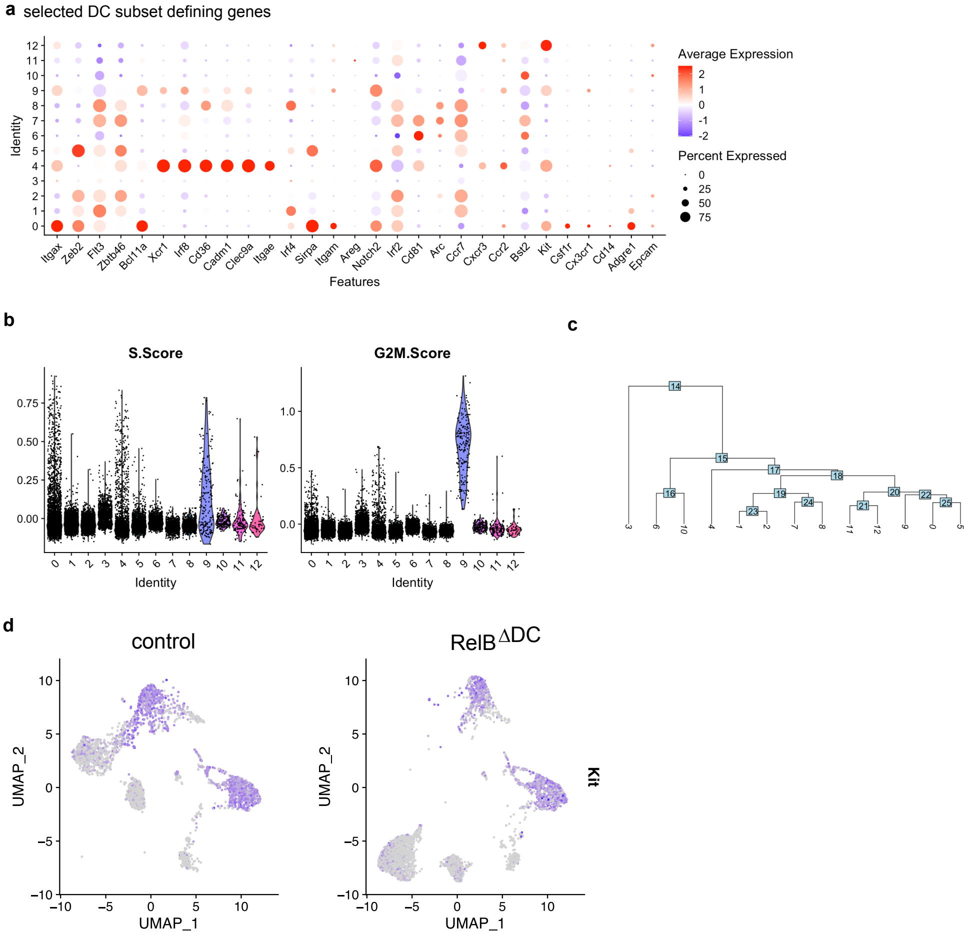

Supplementary Figure 3: Identification of DC subsets in scRNASeq clusters from mLN

Characterization of clusters shown in UMAP of scRNASeq of $CD11c^{+}MHCII^{+}$ cells from mesenteric lymph node (mLN) of control and $RelB^{\Delta DC}$ mice in **Fig. 3a.** **a** Feature plot of cDC, DC1, DC2, migratory DC, pDC, monocyte and fibroblast specific expression levels in indicated clusters. **b** S score and G2M score of indicated clusters. **c** Cluster tree analysis of identified clusters in **Fig. 3a**. **d** UMAP plot depicting *Kit* expression in cDCs from mLN of control and $RelB^{\Delta DC}$ mice.

Supplementary figure 4

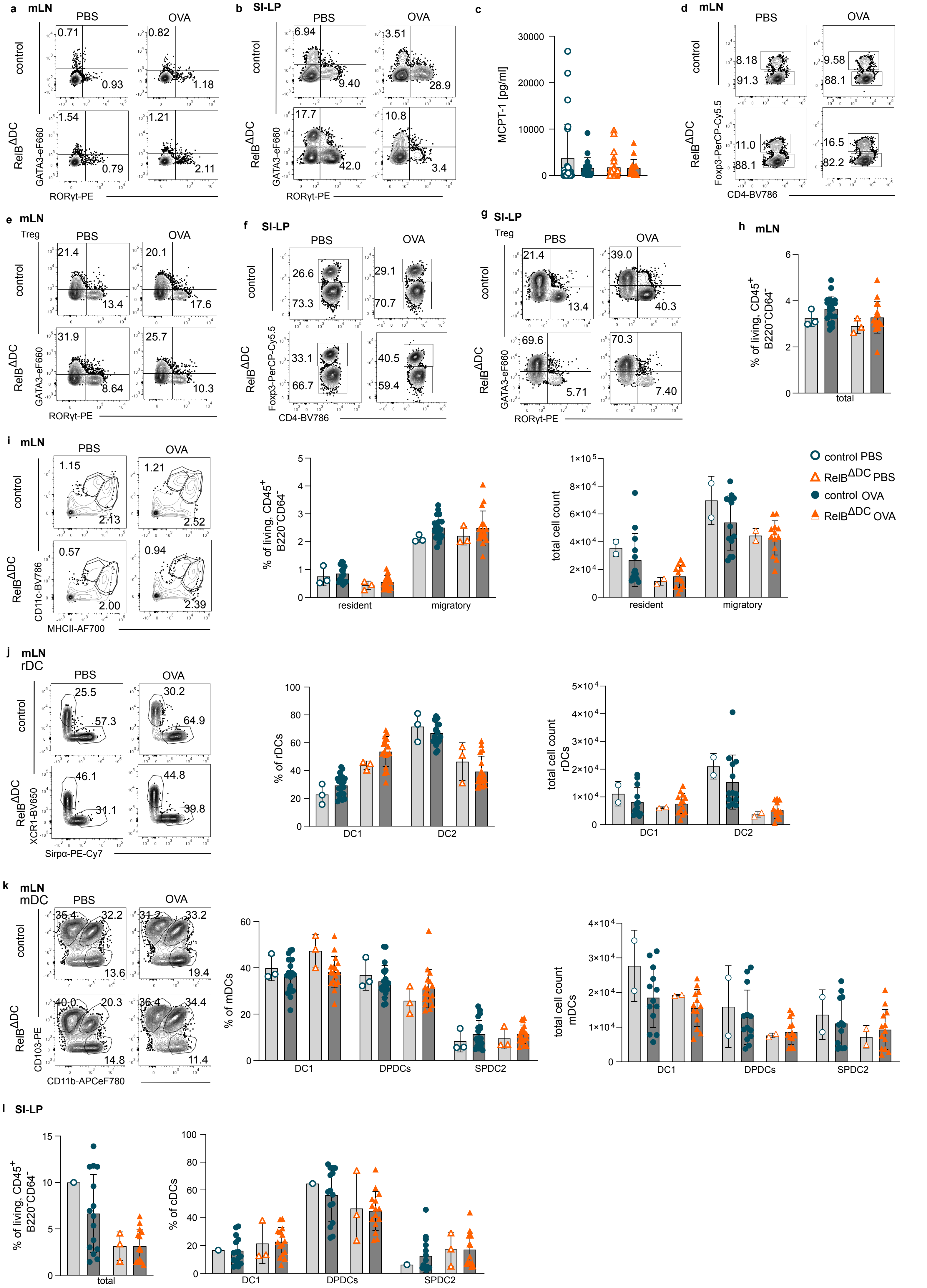

Supplementary Figure 4: Flow cytometric analysis and enumeration of immune cell populations after food allergy

a, b Representative counter plots of GATA3^{+} and RORγt^{+} Foxp3^{-} Th cells in mesenteric lymph node (mLN) (**a**) and lamina propria of the small intestine (SI-LP) (**b**) in control and RelB$^{\Delta DC}$ mice ±OVA on day 36 after induction of cholera toxin driven food allergy. **c** Serum MCPT-1 levels in control and RelB$^{\Delta DC}$ mice ±OVA on day 36 after induction of cholera toxin driven food allergy (control(before) *n* = 26, control(OVA) *n* = 18, RelB$^{\Delta DC}$(before) *n* = 23 and RelB$^{\Delta DC}$(OVA) *n* = 17) **d-g** Flow cytometric analysis of Treg cells in control and RelB$^{\Delta DC}$ ± OVA. **d, e** Representative contour plots of Foxp3 expressing Treg cells (**d**) and GATA3 and RORγt expression in Treg cells (**e**) in mLN. **f, g** Representative contour plots of Foxp3 expressing Treg cells (**f**) and GATA3 and RORγt expression in Tregs (**g**) in SI-LP. **h** Frequency of total CD11c^{high}MHCII^{high} cells (cDCs) out of live/dead^{-}CD45^{+}CD45R^{-}CD64^{-} cells from mLN of control and RelB$^{\Delta DC}$ mice ±OVA on day 36 of cholera toxin driven food allergy (related to **Fig. 4**, control(PBS) *n* = 3, control(OVA) *n* = 20, RelB$^{\Delta DC}$(PBS) *n* = 3 and RelB$^{\Delta DC}$(OVA) *n* = 17). **i-l** Flow cytometric analysis of DC subsets in control and RelB$^{\Delta DC}$ mice ±OVA **i** Representative contour plots (left), quantification of frequency (middle) and total numbers (right) of resident (rDC) and migratory (mDC) DC subsets in mLN (control(PBS) *n*= 3/2, control(OVA) *n* = 20/13, RelB$^{\Delta DC}$(PBS) *n* = 3/2 and RelB$^{\Delta DC}$(OVA) *n* = 17/13). **j** Representative contour plots (left), quantification of frequency (middle) and total numbers (right) of resident DC subsets (DC1 and DC2) in mLN (control(PBS) *n* = 3/2, control(OVA) *n* = 20/13, RelB$^{\Delta DC}$(PBS) *n* = 3/2 and RelB$^{\Delta DC}$(OVA) *n* = 17/13). **k** Representative contour plots (left), quantification of frequency (middle) and total numbers (right) of migratory DC subsets (DC1, DPDC2 and SPDC2) in mLN (control(PBS) *n* = 3/2, control(OVA) *n* = 20/13, RelB$^{\Delta DC}$(PBS) *n* = 3/2 and RelB$^{\Delta DC}$(OVA) *n* = 17/13). **l** Frequency of total CD11c^{high}MHCII^{high} cells (cDCs) out of live/dead^{-}CD45^{+}CD45R^{-}CD64^{-} cells (left) and DC1, DPDC2 and SPDC2 (right) from SI-LP of control and RelB$^{\Delta DC}$ mice ±OVA on day 36 of cholera toxin driven food allergy (control(PBS) *n* = 1, control(OVA) *n* = 15, RelB$^{\Delta DC}$(PBS) *n* = 3 and RelB$^{\Delta DC}$(OVA) *n* = 15). Each dot represents an individual mouse and mean ± SD from two to four independent experiments is shown. Statistical analysis was performed using two-tailed students t-test. P value of <0.05 was considered statistically significant with *p <0.05, **p< 0.01, ***p < 0.001, ****p <0.0001. Source data are provided as a Source Data file.

Supplementary figure 5

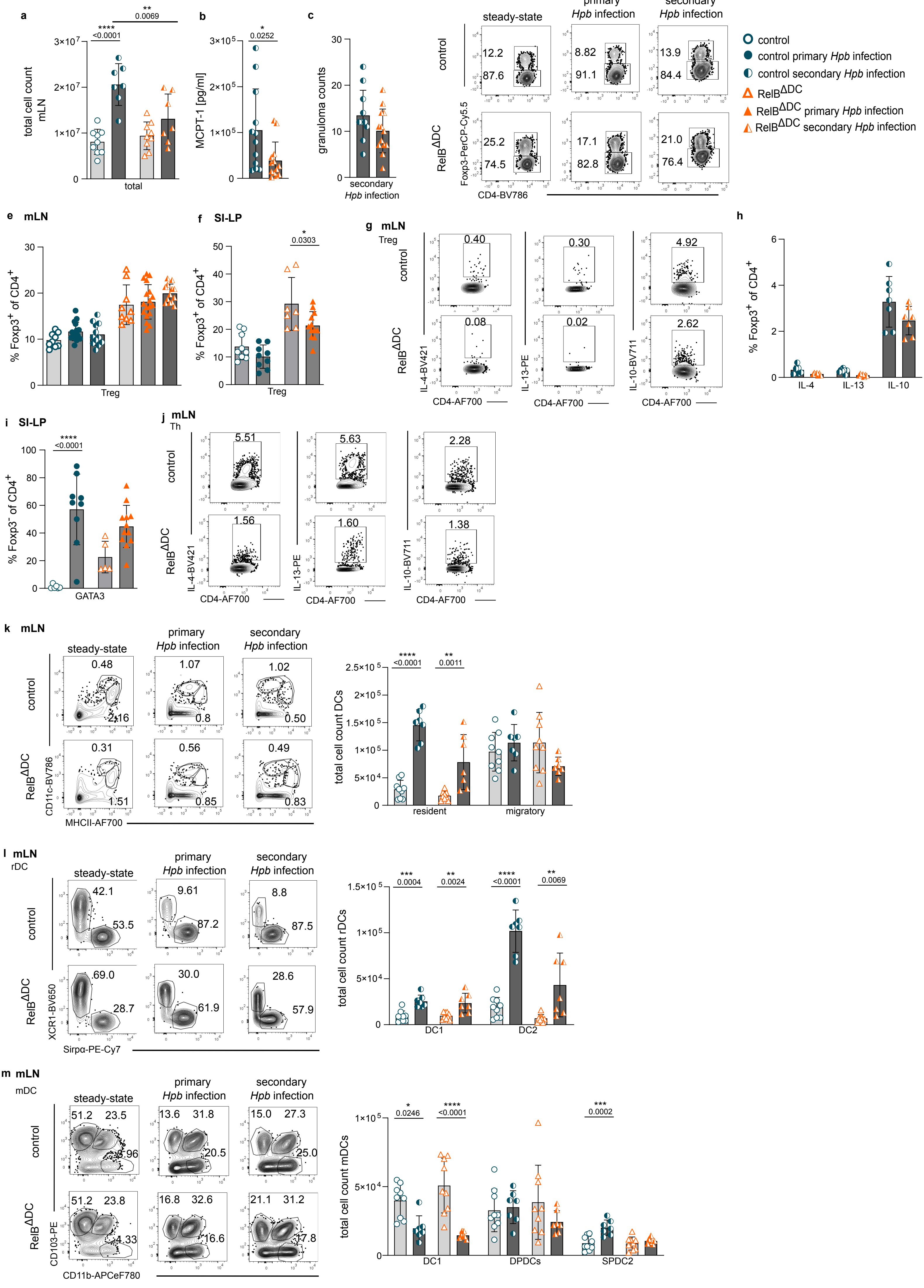

Supplementary Figure 5: Flow cytometric analysis and enumeration of immune cells after primary and secondary *Hpb* infection

a Total cell number of living CD45$^+$ cells from mesenteric lymph node (mLN) of control and RelB$^{\Delta DC}$ mice at steady state and on day 63 after secondary *Heligmosomoides polygyrus bakeri* (*Hpb)* infection (control (steady state) *n* = 9, control (secondary *Hpb* infection) *n* = 7, RelB$^{\Delta DC}$ (steady state) *n* = 9 and RelB$^{\Delta DC}$(secondary *Hpb* infection) *n* = 7. **b** Serum MCPT-1 levels in control and RelB$^{\Delta DC}$ mice after secondary *Hpb* infection (day 63, related to **Fig. 5**, control (secondary *Hpb* infection) *n* = 12, RelB$^{\Delta DC}$(secondary *Hpb* infection) *n* = 13). **c** Intestinal granuloma counts of small intestinal tissue sections after secondary *Hpb* infection (related to **Fig. 5d**, control (secondary *Hpb* infection) *n* = 11, RelB$^{\Delta DC}$(secondary *Hpb* infection) *n* = 13). **d, e** Representative contour plots (**d**) and quantification (**e**) of Foxp3 expressing Th cell frequencies from mLN at steady state, after primary and after secondary *Hpb* infection (control (steady state) *n* = 9 control (primary *Hpb* infection) *n* = 15 and control (secondary *Hpb* infection) *n* = 12. RelB$^{\Delta DC}$ (steady state) *n* = 9, RelB$^{\Delta DC}$(primary *Hpb* infection) *n* = 18, and RelB$^{\Delta DC}$(secondary *Hpb* infection) *n* = 13. **f** Quantification of Foxp3 expressing Th cell frequencies from SI-LP at steady state and after secondary *Hpb* infection (control (steady state) *n* = 9, control (primary *Hpb* infection) *n* = 9, RelB$^{\Delta DC}$ (steady state) *n* = 7, RelB$^{\Delta DC}$(primary *Hpb* infection) *n* = 12). **g, h** Representative contour plots (**g**) and total quantification (**h**) of IL-4, IL-13 and IL-10 producing Foxp3$^+$ Treg cell frequencies in mLN from control and RelB$^{\Delta DC}$ mice after secondary *Hpb* infection (control (secondary *Hpb* infection) *n* = 7, RelB$^{\Delta DC}$(secondary *Hpb* infection) *n* = 7). **i** Quantification of GATA3-expressing Foxp3$^+$ Treg cell frequencies in SI-LP at steady state and after primary *Hpb* infection (control (steady state) *n* = 6 control (primary *Hpb* infection) *n* = 9, RelB$^{\Delta DC}$ (steady state) *n* = 5, RelB$^{\Delta DC}$(primary *Hpb* infection) *n* = 10). **j** Representative contour plots of IL-4, IL-13 and IL-10 producing Foxp3$^-$ T helper cells in mLN from control and RelB$^{\Delta DC}$ mice after secondary *Hpb* infection. **k-m** Flow cytometric analysis of DC subsets in mLN of control and RelB$^{\Delta DC}$ mice at steady state, after primary and secondary *Hpb* infection. **k** Representative contour plots of resident and migratory DC at steady state, after primary and secondary *Hpb* infection (left) and total cell numbers of DCs at steady state and after secondary *Hpb* infection (right) (control (steady state) *n* = 9, control (secondary *Hpb* infection) *n* = 7, RelB$^{\Delta DC}$ (steady state) *n* = 9, RelB$^{\Delta DC}$(secondary *Hpb* infection) *n* = 7). **l** Representative contour plots of resident DC subsets (DC1 and DC2) at steady state, after primary and secondary *Hpb* infection (left) and total cell numbers at steady state and after secondary *Hpb* infection (right) (control (steady state) *n* = 9, control (secondary *Hpb* infection) *n* = 7, RelB$^{\Delta DC}$ (steady state) *n* = 9, RelB$^{\Delta DC}$(secondary *Hpb* infection) *n* = 7). **m** Representative contour plots of migratory (mDCs) DC subsets (DC1, DPDC2 and SPDC2) at steady state, after primary and secondary *Hpb* infection (left) and total cell numbers at steady state and after secondary *Hpb* infection (right) (control (steady state) *n* = 9, control (secondary *Hpb* infection) *n* = 7, RelB$^{\Delta DC}$ (steady state) *n* = 9, RelB$^{\Delta DC}$(secondary *Hpb* infection) *n* = 7). Each dot represents an individual mouse and mean ± SD from at least two independent experiments is shown. Statistical analysis was performed using two-tailed students t-test (**b**, **c**, **f**, **h**, **i**, **k-m**) or one-way ANOVA with Tukey correction for multiple comparison (**a**, **e**). P value of <0.05 was considered statistically significant with *p <0.05, **p< 0.01, ***p < 0.001, ****p <0.0001. Source data are provided as a Source Data file.

Supplementary figure 6

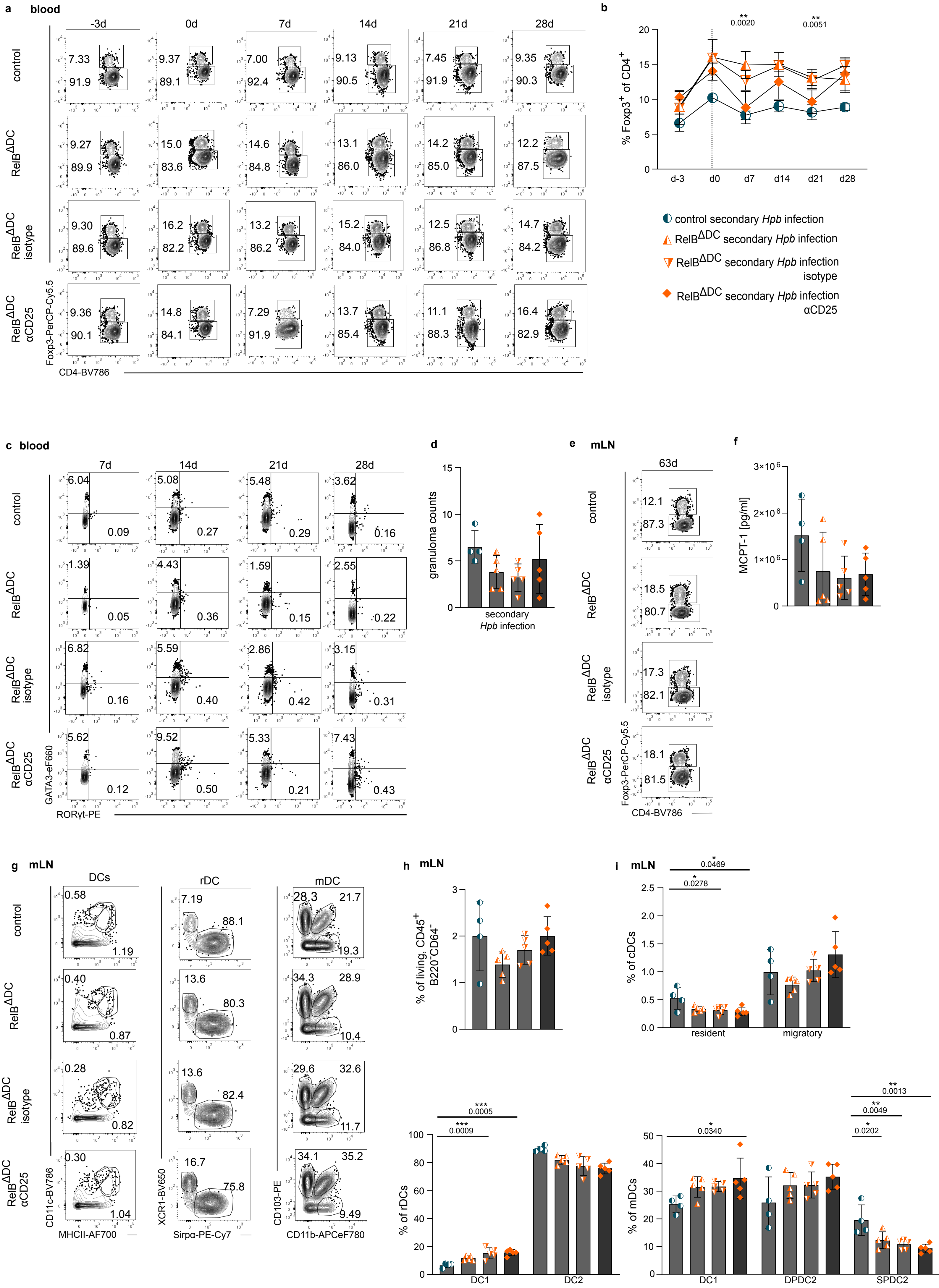

Supplementary Figure 6: Flow cytometric analysis and enumeration of immune cell populations in secondary *Hpb* infection in context of anti-CD25 antibody treatment

a, b Flow cytometric analysis of Treg populations in blood after primary *Heligmosomoides polygyrus bakeri* (*Hpb)* infection in control and RelB$^{\Delta DC}$ mice ± anti-CD25/isotype control antibody treatment. Representative contour plots (**a**) of Foxp3^{+} expression and quantification (**b**) in Th cell frequencies at indicated timepoints after primary *Hpb* infection. **c** Representative contour plots of GATA3 and RORγt expression in Foxp3^{-} Th cells at indicated timepoints after primary *Hpb* infection. **d** Quantification of intestinal granuloma counts of small intestinal tissue section after secondary *Hpb* infection. **e** Representative contour plots of Foxp3 expressing T cells at day 63 after secondary *Hpb* infection. **f** Serum MCPT-1 levels in control mice, RelB$^{\Delta DC}$ mice and RelB$^{\Delta DC}$ mice treated with isotype- or anti-CD25 antibody on day 63 after secondary *Hpb* infection. **g-i** Flow cytometric analysis of DC subsets in mesenteric lymph node (mLN) after secondary *Hpb* infection of control mice, RelB$^{\Delta DC}$ mice and RelB$^{\Delta DC}$ mice treated with isotype- or anti-CD25 antibody treated RelB$^{\Delta DC}$ mice on day 63 after secondary *Hpb* infection. **g** Representative contour plots of DC subsets (resident DC1 and DC2, migratory DC1, DPDC2 and SPDC2) in mLN. **h** Frequency of total cDCs (CD64^{-}B220^{-}CD11c^{high}MHCII^{high}) in mLN. **i** Quantification of frequency of resident (rDCs) and migratory (mDCs) DCs (top), resident DC subsets (DC1 and DC2) (bottom left) and migratory DC subsets (DC1, DPDC2 and SPDC2) (bottom right) of control mice, RelB$^{\Delta DC}$ ± anti-CD25/isotype control antibody treatment at day 63 after secondary *Hpb* infection. Each dot represents an individual mouse and mean ± SD from one experiment is shown. control *n* = 4, RelB$^{\Delta DC}$ *n* = 5, RelB$^{\Delta DC}$(αCD25) *n* = 5, RelB$^{\Delta DC}$(isotype) *n* = 5. Statistical analysis was performed using One-Way-ANOVA with Tukey correction for multiple comparison (**d**, **f**, **h**, **i**) for **b**, untreated RelB$^{\Delta DC}$ mice were compared to anti-CD25 treated RelB$^{\Delta DC}$ mice with two-tailed students t-test. P value of <0.05 was considered statistically significant with *p <0.05, **p< 0.01, ***p < 0.001, ****p <0.0001. Source data are provided as a Source Data file.

Supplementary figure 7

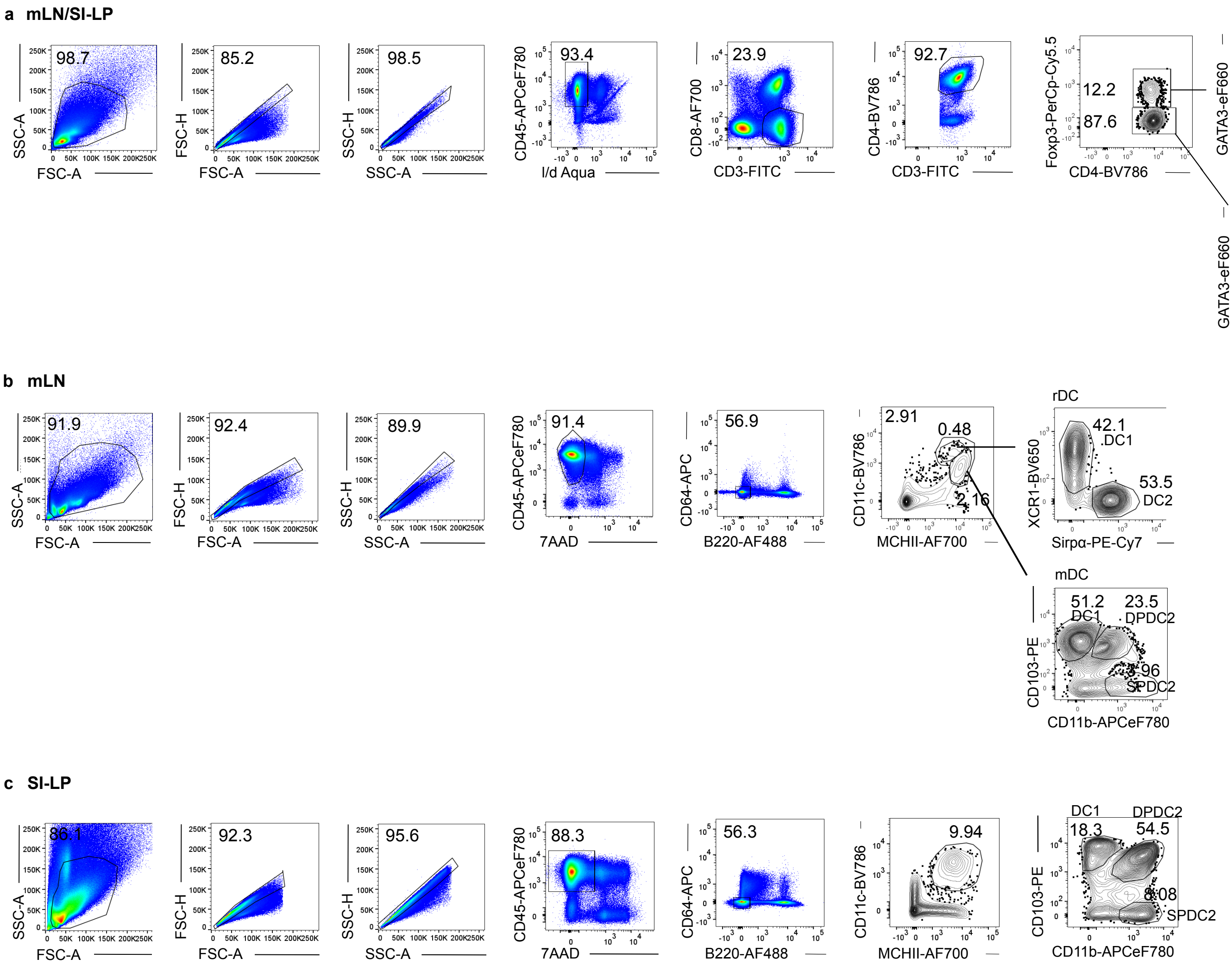

Supplementary Figure 7: Gating strategy for the identification of T cells and cDCs

a Representative flow cytometry plots showing gating strategy for the identification of $Foxp3^+$ Tregs, $Foxp3^-$ Th cells and their expression of GATA3 and RORγt in mesenteric lymph node (mLN). Same strategy was used for T cells in lamina propria of the small intestine (SI-LP). **b** Representative flow cytometry plots showing the gating strategy for the identification of cDCs, resident DCs (rDCs), migratory DCs (mDCs), resident DC subsets and migratory DC subsets in mLN. **c** Representative flow cytometry plots showing gating strategy for the identification of cDCs and their subsets in SI-LP.
