# Supplementary material for: Dominant immune tolerance in the intestinal tract imposed by RelB-dependent migratory dendritic cells regulates protective type 2 immunity
Source: Nat Commun. 2024 Oct 23;15:9143. doi: 10.1038/s41467-024-53112-9 (PMC11500181; doi:10.1038/s41467-024-53112-9)
Supplement: Supplementary file 2 — Reporting Summary [file 41467_2024_53112_MOESM2_ESM.pdf]

nature portfolio

Corresponding author(s): Caspar Ohnmacht

Last updated by author(s): Sep 25, 2024

Reporting Summary

Nature Portfolio wishes to improve the reproducibility of the work that we publish. This form provides structure for consistency and transparency in reporting. For further information on Nature Portfolio policies, see our Editorial Policies and the Editorial Policy Checklist.

Statistics

For all statistical analyses, confirm that the following items are present in the figure legend, table legend, main text, or Methods section.

n/a	Confirmed	
☐	☒	The exact sample size (*n*) for each experimental group/condition, given as a discrete number and unit of measurement
☐	☒	A statement on whether measurements were taken from distinct samples or whether the same sample was measured repeatedly
☐	☒	The statistical test(s) used AND whether they are one- or two-sided<br>*Only common tests should be described solely by name; describe more complex techniques in the Methods section.*
☒	☐	A description of all covariates tested
☐	☒	A description of any assumptions or corrections, such as tests of normality and adjustment for multiple comparisons
☐	☒	A full description of the statistical parameters including central tendency (e.g. means) or other basic estimates (e.g. regression coefficient) AND variation (e.g. standard deviation) or associated estimates of uncertainty (e.g. confidence intervals)
☐	☒	For null hypothesis testing, the test statistic (e.g. *F*, *t*, *r*) with confidence intervals, effect sizes, degrees of freedom and *P* value noted<br>*Give P values as exact values whenever suitable.*
☒	☐	For Bayesian analysis, information on the choice of priors and Markov chain Monte Carlo settings
☒	☐	For hierarchical and complex designs, identification of the appropriate level for tests and full reporting of outcomes
☒	☐	Estimates of effect sizes (e.g. Cohen's *d*, Pearson's *r*), indicating how they were calculated

Our web collection on statistics for biologists contains articles on many of the points above.

Software and code

Policy information about availability of computer code

Data collection	-flow cytometry data was colletced using BD FACSDIVA Software (BD BioSciences, Version 8.0.1)<br>- total cell counts were collected using BD Accuri C6 (BD BioSciences, Version 1.0.264.21)<br>- Imaging data was collected using AxioScan 7 (Zeiss)
Data analysis	- FlowJo software (Tree Star, Inc.), Version 10 was used for analysis of flow cytometry data<br>- Microsoft Excel (Version 2403) was used for table creation<br>- Microsoft PowerPoint (Version 2430) was used for experimental setup<br>- GraphPad Prism 10 was used for graphical and statistical analysis<br>- for single cell RNA sequenzing, data was processed using Cellranger (version 7.0.1) for mouse genome alignment (10x genomics reference build MM10 2020 A). Subsequent data processing was performed in R (Version 4.3) using Seurat (Version 4.3) as described in Material and Methods<br>- LEGENDplex cloud based program was used for analysis of cytokines in culture supernatants<br>- ZEISS ZEN (Version 3.7) was used for analysis of image data

For manuscripts utilizing custom algorithms or software that are central to the research but not yet described in published literature, software must be made available to editors and reviewers. We strongly encourage code deposition in a community repository (e.g. GitHub). See the Nature Portfolio guidelines for submitting code & software for further information.

Data

Policy information about availability of data

All manuscripts must include a data availability statement. This statement should provide the following information, where applicable:

- Accession codes, unique identifiers, or web links for publicly available datasets
- A description of any restrictions on data availability
- For clinical datasets or third party data, please ensure that the statement adheres to our policy

The authors declare that the data supporting the findings of this study are available within the paper and its Supplementary Files. SOurce data are provided with this paper in the Source data file. Raw data are available on request. Raw Datasets related to single cell sequencing experiments that were generated and analyzed for the current study have been deposited and made publicly available in the Gene Expression Omnibus under the accession number GSE243483 (https://www.ncbi.nlm.nih.gov/geo/query/acc.cgi?acc=GSM7789315).

Research involving human participants, their data, or biological material

Policy information about studies with human participants or human data. See also policy information about sex, gender (identity/presentation), and sexual orientation and race, ethnicity and racism.

Reporting on sex and gender	n/a
Reporting on race, ethnicity, or other socially relevant groupings	n/a
Population characteristics	n/a
Recruitment	n/a
Ethics oversight	n/a

Note that full information on the approval of the study protocol must also be provided in the manuscript.

Field-specific reporting

Please select the one below that is the best fit for your research. If you are not sure, read the appropriate sections before making your selection.

☒ Life sciences ☐ Behavioural & social sciences ☐ Ecological, evolutionary & environmental sciences

For a reference copy of the document with all sections, see nature.com/documents/nr-reporting-summary-flat.pdf

Life sciences study design

All studies must disclose on these points even when the disclosure is negative.

Sample size	No statistical methods were used to predetermine the sample size. Most experiments were repeated at least 2 times to ensure an overall sample size of at least 3 per experimental group. The exact n values used to calculate the statistics are provided per experiment presented in the main and supplementary figure legends of the manuscript. For single cell RNA sequenzing, cells from three mice were pool for each group in order to get sufficient numbers of cells and avoid cell-isolation biases. For anti-CD25 treatment, due to animal warefare issues, only one experiment was performed. According to the 3R principle, we chose the minimal replicate number sufficient to ascertain statistics by unpaired t-test or one-way ANOVA.
Data exclusions	For single cell RNA sequenzing, the following criteria were used for quality control: a) more than 200 genes detected b) less than 20% mitochondrial genes reads c) more than 5% ribosomal protein genes reads d) less than 20% hemoglobin genes reads e) singlets as determined by doubletFinder (version 2.0.3, pK = 0.09, PCs=1:10). Only genes detected in at least 4 cells were kept.<br>In general, no data was excluded from analysis except for poor cell isolation efficiency determined for analysis of flow cytometric data. Sample quality was determined by a sufficient amount of isolated cells, proper dead cell/living cell ratio and a clearly recognizable population of CD45 + cells. This was especially crucial for cells isolated from small intestine in context of inflammation.
Replication	Number of replicates is indicated in each Figure legend. In general, data presented are the result of at least 2 independent experiments with multiple biological replicates. All attempts for replication were successful.
Randomization	Mice were housed in the same room and rack in the respective animal facility and assigned randomly to experimenatl groups. Sex- and age-matched littermates were used for experiments whenever possible. For single cell RNA sequencing experiments mice were age and sex-matched.
Blinding	The investigators were blinded during treatment of mice with indicated agents. Counting of Hpb- worms during infection and analysis of granuloma counts in histologic samples was performed in a blinded manner. The investigators were not blinded during collection of animal tissues due to requirements for cage identification and labeling for treatment purposes.<br>In all experiments, samples were processed simultaneously or in parallel.

Reporting for specific materials, systems and methods

We require information from authors about some types of materials, experimental systems and methods used in many studies. Here, indicate whether each material, system or method listed is relevant to your study. If you are not sure if a list item applies to your research, read the appropriate section before selecting a response.

Materials & experimental systems

n/a	Involved in the study
☐	☒ Antibodies
☒	☐ Eukaryotic cell lines
☒	☐ Palaeontology and archaeology
☐	☒ Animals and other organisms
☒	☐ Clinical data
☒	☐ Dual use research of concern
☒	☐ Plants

Methods

n/a	Involved in the study
☐	☐ ChIP-seq
☐	☒ Flow cytometry
☐	☐ MRI-based neuroimaging

Antibodies

Antibodies used

All antibody details are listed in Material and Methodes and also listed below:
Antigen-Clone-Fluorochrome-Source-Catalog number:

CD103 2E7 PE BioLegend 121406 1:100
CD117 2B8 PerCP/Cy5.5 BioLegend 105824 1:100
CD11b M1/70 APC-e780 eBiosciences 47-0112-82 1:100
CD11c N418 BV786 BioLegend 117335 1:200
CD16/CD32 BD Biosciences 553142 1:300
CD172a P84 PE-Cy7 BioLegend 144008 1:100
CD200 OX-90 APC BioLegend 123180 1:100
CD3e 145-2C11 FITC BD Biosciences 553062 1:100
CD3e 17A2 AF700 BioLegend 100216 1:100
CD4 GK1.5 BV786 BioLegend 100453 1:400
CD4 RM4-5 AF700 BD Biosciences 557956 1:400
CD44 IM7 APC-e780 eBiosciences 47-0441-80 1:200
CD45 30-F11 APC-e780 eBiosciences 47-0451-82 1:100
CD45 30-F11 FITC eBiosciences 11-0451-82 1:500
CD45.2 104 PB BioLegend 109820 1:200
CD45R RA3-6B2 AF488 BioLegend 103225 1:200
CD63 NVG-2 APC BioLegend 143906 1:100
CD64 X54-5/7.1 APC BioLegend 139306 1:100
CD8a 53-6.7 AF700 eBiosciences 56-0081-82 1:100
CD8a 5H10 APC-e780 Invitrogen 47-4321-82 1:600
Foxp3 FJK-16s PerCP-Cy5.5 eBiosciences 45-5773-82 1:100
GATA3 TWAJ eF660 eBiosciences 50-9966-42 1:20
IL-10 JES5-16E3 BV711 BD Biosciences 564081 1:100
IL-13 eBio13A PE eBiosciences 12-7133-82 1:100
IL-4 11B11 BV421 BD Biosciences 562915 1:100
KLRG-1 2F1 BV421 BioLegend 138414 1:100
MHCII M5/114.15.2 AF700 BioLegend 107622 1:300
PD-1 29F.1A12 BV785 BioLegend 135225 1:100
Plet-1 custom-made Biotin obtained from C. Ruedl
RORgt AFKJS-9 PE eBiosciences 12-6988-82 1:100
ST-2(IL33R) RMST2-2 Biotin eBiosciences 13-9335-82 1:100
XCR1 ZET BV650 BioLegend 148220 1:200

Streptavidin PE BD Biosciences 554061 1:1500
Streptavidin PE -Cy7 BioLegend 405206 1:1000

Antibody used for in vivo targeting-Clone-Source-Catalog number:
anti-CD25 antibody PC-61.5.3 BioXCell BP0012
isotype control antibody HRPN BioXCell BP0088

Validation	Commercially available antibodies have been validated by their respective vendors for species reactivity and application in flow cytometry. Validation data are available on the manufacturer's website using the catalog number of each product.<br>Single color controls and Fluorescence Minus One Controls were used to ensure high quality data.<br><br>Plet-1 antibody was used according to PMID: 33207209<br><br>Antibodies used for in vivo targeting experiments were used according to PMC6826119

Animals and other research organisms

Policy information about studies involving animals; ARRIVE guidelines recommended for reporting animal research, and Sex and Gender in Research

Laboratory animals	Age- and sex-matched male and female mice of 8 - 14 weeks of age (unless the duration of the experiment required a longer lifespan) were used and Cre- littermates were chosen as control animals whenever possible. Mice were euthanized by cervical dislocation.<br><br>For this study, following mouse strains were bred at the Helmholtz Zentrum Munich in specific pathogen-free conditions:<br>- B6.Cg-Tg(Itgax-cre)1–1Reiz/Jmice (CD11c-cre) (The Jackson Laboratory; strain #008068)<br>- RelBfl/fl (DOI: 10.1016/j.jaut.2017.03.007)<br>- CD11c-cre mice crossbred with RelB fl/fl mice: RelBdDC mice in the manuscript<br><br>LtbRdDC mice were provided by Prof. Dr. Andreas Diefenbach (PMID: 33207209)<br><br>Mice were maintained in specific pathogen-free SPF conditions with a 12 hour dark/light cycle, in individually vented cages (IVCs, type II long cages, measuring 18x30x13 cm with stocking density according to EU guideline 2010/63) supplied with autoclaved bedding, play tunnels, nestles and mouse houses. Irradiated food and sterile filtered and UV-light exposed water were provided ad libitum. Cage manipulations took place in laminar flow hoods. Air temperature was 22 ± 2°C and humidity 55 ± 10% with daily control and record.
Wild animals	No wild animals were used in this study.
Reporting on sex	Mice were age- and sex-matched but male and female mice were used for all experiments.
Field-collected samples	No field-collected samples were used in this study
Ethics oversight	All interventions were performed in accordance with the European Convention for Animal Care and Use of Laboratory Animals and were approved by government authorities (Regierung von Oberbayern, license numbers ROB55.2-2532.Vet_02-15-5, ROB55.2-2532.Vet_02-17-222 and ROB55.2-2532.Vet_02-21-48).

Note that full information on the approval of the study protocol must also be provided in the manuscript.

Plants

Seed stocks	*Report on the source of all seed stocks or other plant material used. If applicable, state the seed stock centre and catalogue number. If plant specimens were collected from the field, describe the collection location, date and sampling procedures.*
Novel plant genotypes	*Describe the methods by which all novel plant genotypes were produced. This includes those generated by transgenic approaches, gene editing, chemical/radiation-based mutagenesis and hybridization. For transgenic lines, describe the transformation method, the number of independent lines analyzed and the generation upon which experiments were performed. For gene-edited lines, describe the editor used, the endogenous sequence targeted for editing, the targeting guide RNA sequence (if applicable) and how the editor was applied.*
Authentication	*Describe any authentication procedures for each seed stock used or novel genotype generated. Describe any experiments used to assess the effect of a mutation and, where applicable, how potential secondary effects (e.g. second site T-DNA insertions, mosiacism, off-target gene editing) were examined.*

ChIP-seq

Data deposition

☐ Confirm that both raw and final processed data have been deposited in a public database such as GEO.

☐ Confirm that you have deposited or provided access to graph files (e.g. BED files) for the called peaks.

Data access links<br>*May remain private before publication.*	*For "Initial submission" or "Revised version" documents, provide reviewer access links. For your "Final submission" document, provide a link to the deposited data.*
Files in database submission	*Provide a list of all files available in the database submission.*

Genome browser session
(e.g. UCSC)

Provide a link to an anonymized genome browser session for "Initial submission" and "Revised version" documents only, to enable peer review. Write "no longer applicable" for "Final submission" documents.

Methodology

Replicates

Describe the experimental replicates, specifying number, type and replicate agreement.

Sequencing depth

Describe the sequencing depth for each experiment, providing the total number of reads, uniquely mapped reads, length of reads and whether they were paired- or single-end.

Antibodies

Describe the antibodies used for the ChIP-seq experiments; as applicable, provide supplier name, catalog number, clone name, and lot number.

Peak calling parameters

Specify the command line program and parameters used for read mapping and peak calling, including the ChIP, control and index files used.

Data quality

Describe the methods used to ensure data quality in full detail, including how many peaks are at FDR 5% and above 5-fold enrichment.

Software

Describe the software used to collect and analyze the ChIP-seq data. For custom code that has been deposited into a community repository, provide accession details.

Flow Cytometry

Plots

Confirm that:

- [x] The axis labels state the marker and fluorochrome used (e.g. CD4-FITC).
- [x] The axis scales are clearly visible. Include numbers along axes only for bottom left plot of group (a 'group' is an analysis of identical markers).
- [x] All plots are contour plots with outliers or pseudocolor plots.
- [x] A numerical value for number of cells or percentage (with statistics) is provided.

Methodology

Sample preparation

Isolation of immune cells:
Steady-state lamina propria of the small intestine
Single cell suspensions from the lamina propria of small intestine were prepared based on a slightly modified protocol previously described in PMID 26160380. Briefly, the small intestine was removed, placed in ice-cold DPBS (Gibco) and Peyer's patches were removed. Intestines were cut open longitudinally, washed with ice-cold PBS, and cut into 0.5 - 1 cm pieces. For mucus removal, small intestine pieces were incubated in RPMI 1640 (Gibco) containing 25 mM HEPES (Gibco), 5 mM EDTA (Invitrogen), 3% FCS (Sigma Aldrich), and 0.145 mg/ml DTT (Sigma) for 20 min at 37°C, 80 rpm. Thereafter, tissues were washed three times with RPMI1640 containing 25 mM HEPES and 2 mM EDTA and minced with sharp scissors into 1-2 mm pieces. To generate of single cell suspensions, minced pieces were digested for 30 min by shaking at 100 rpm at 37°C in RPMI 1640 containing 25 mM HEPES, 200 iU/ml Collagenase IV (Worthington), and 0.2 mg/ml DNaseI (Sigma). Tissue pieces were then pipetted up and down several times and cell suspensions were collected. One more cycle of digestion with fresh medium was performed and cell suspensions were filtered through a 100 µm cell strainer (Corning). Cell suspension was centrifuged at 450 g, for 5 min at 4°C, cell pellets were resuspended in 40% Percoll (Cytiva) and layered onto an 80% Percoll solution. The Percoll gradient was centrifuged at 1600 g at room temperature for 15 min. The interlayer containing lamina propria mononuclear cells was collected in FACS buffer (DPBS containing 1% FCS, 25 mM HEPES, and 2.5 mM EDTA). This single cell suspension was then used for downstream analysis.

Lamina propria of the small intestine after Hpb infection:
Isolation of lamina propria cells from Hpb-infected mice was performed using an alternative cell isolation protocol, adjusted to a high inflammatory status and mucus production. Small intestinal tissues were harvested as described before, but for mucus removal, tissue pieces were incubated in HBSS (Gibco) containing 2 mM EDTA for 10 min at 37°C with shaking at 200 rpm, and afterward pulse vortexed three times for 5 sec at 2500 rpm. The supernatant was discarded and fresh HBSS containing 2 mM EDTA was added. This process was repeated four times. For digestion, washed small intestinal pieces were minced and incubated two times with RPMI 1640 containing 20% FCS, 0.2 mg/ml DNaseI, and 1 mg/ml Collagenase A (Roche) for 15 min at 37°C and shaking at 200 rpm. Cell suspensions were filtered through a 100 µm strainer, collected in FACS buffer, and stored on ice. Percoll gradient was performed as described for the steady-state protocol.

Lymphoid organs
To generate single cell suspensions from lymphoid organs, spleen and indicated lymph nodes were collected in RPMI1640 containing 25 mM HEPES and 5% FCS, and tissues were minced into 1-2 mm pieces. Tissue pieces were digested by incubation in RPMI 1640 containing 25 mM HEPES, 200 iU/ml Collagenase IV (Worthington), and 0.2 mg/ml DNAseI (Sigma) for 30 min at 37°C, shaking at 100 rpm. Cell suspensions were filtered through a 70 µm cell strainer, collected in FACS buffer and centrifuged at 450 g for 5 min at 4°C. For spleen, erythrocytes were removed by incubating cells in 2 ml of ACK lysis buffer (0.15 M ammonium chloride (Sigma), 10 mM potassium hydrogen carbonate (Merck), 1 mM EDTA-di sodium, pH-adjusted to 7.3) for 2 min, before samples were washed and resuspended with respective buffers for further analysis.

Only exception was determination of CD117 levels, for which mLN samples were treated equally but digestion with enzymes was skipped to ensure CD117 molecule integrity.

Extracellular staining
Cell numbers were routinely counted with an Accuri C6 Flow Cytometer (BD). Single-cell suspensions were first incubated for 10 min at 4°C with an Fc blocking antibody in FACS buffer (CD16/CD32, BD), followed by 20 min incubation at 4°C with the indicated antibodies in FACS buffer. 7-Aminoactinomycin D (7-AAD) (Enzo) or fixable Zombie Aqua (BioLegend) was used for live/dead cell discrimination according to the manufacturer's instructions.

Intracellular staining of transcription factors
Intracellular staining of transcription factors was performed with a Foxp3 fixation/permeabilization kit (ThermoFisher) according to the manufacturer's instructions. Fixed cells were intracellularly stained with the indicated antibodies for 60 min at room temperature.
Intracellular staining of cytokines

For staining of intracellular cytokines, 3x106 cells in single-cell suspension were seeded in U-bottom 96-well plates (Sarstedt). Cells were incubated in complete RPMI (RPMI 1640, 2 mM L-Glutamine (Gibco), 10000 U Penicillin G and 10 mg/ml Streptomycin (Gibco), 10% FCS, 50 µM β-mercaptoethanol (Sigma)) containing 200 ng/ml PMA (Sigma) and 1 µg/ml Ionomycin (Cayman) for two hours at 37°C, 5% CO2. Afterwards, 5 µg/ml Brefeldin A (Sigma) was added, and cells were incubated for an additional two hours. Subsequently, cells were harvested, incubated with Fc block, and extracellular staining was performed by incubating samples with indicated antibodies for 20 min at 4°C. For the fixation of samples, the Cytofix/Cytoperm Kit (BD) was used according to the manufacturer's protocol. Intracellular staining was performed overnight at 4°C. Fluorescence minus one (FMO) controls were performed to control the quality of cytokine staining.

Instrument

LSR Fortessa (BD Biosciences), ARIA III Fusion (BD Biosciences), Accuri C6 Flow Cytometer (BD Biosciences)

Software

Data collection: BD FACSDica Software (BD BioSciences)
Data Analysis: FlowJo software (Tree Star, Inc.)

Cell population abundance

abundance post sort purity was typically >95%, purity was confirmed by post sort flow cytometric re-analysis

Gating strategy

For Analysis of T helper cell popluations in mLN and SI-LP, all samples were initially gated on SSC-A vs FSC-A plot, doublet exclution was performed using FSC-H vs FSC-A and SSC-H vs SSC-A. Living, CD45+ lymphocytes were determined by exclution of fixable viability dye fixable Zombie Aqua (BioLegend) or 7-Aminoactinomycin D (7-AAD) (Enzo) cells vs CD45-APC-eF780 positiv cells. CD8-AF700 vs CD3-FITC was used to exclude CD8+ T cells. CD4+ T cells were identified by CD4-BV786 and CD3-FITC staining. By Foxp3-PerCP-Cy5.5 levels, Foxp3+ Tregs were distingished from Foxp3- T helper cells. Both polulations were analysed for their GATA3-eF660 and TATgt-PE populations. Gating strategy is also shown in Supplementary Figure 7a.

Single cell suspensions from mLN were analysed for their DC-populations by gateing on SSC-A vs FSC-A plot, doublet exclution was performed using FSC-H vs FSC-A and SSC-H vs SSC-A. Living, CD45-APC-eF780+ lymphocytes were gated by exlution of 7AAD positive cells and including CD45-APC-eF780 positiv cells. B cell and Macrophage/Monocyte contamination was prefented by gating for CD64-APC negative, B220-AF488 negative cells. total classical dendritic cells were identified by high levels of CD11c-BV786 and MHCII-AF700 staining. within this population, resident DCs were identified by high levels of CD11c-BV786, but intermediate levels of MHCII-AF700. Migratory DCs were determined by intermediate levels of CD11c-BV786, but high levels of MCHII-AF700. resident DC subsetwere gated for XCR1-BV650 (rDC1) and Sirpa-PE-Cy7(rDC2). Migratory DC subsets analysed for CD103-PE positive (mDC1), CD11b-APCeF780 positive (mSPDC2) and CD103-PE CD11b-APCeF780 double positive mDPDC2s. This gating strategy can be found in Supplementary figure 7b.

Dendritic cells in SI-LP were identified in single cell supsension according to mLN until gating for CD11c-BV786 vs MHCII-AF700 cells. These classical DCs were analysed for CD103-PE positive DC1s, CD11b-APC-eF780 positive and CD103-PE, CD11b-APCeF780 double positive DPDC2s. Gating strategy for SI-LP is shown in supplementray figure 7c.

Single colour controls and flourescence minus one were used to ensure proper compensation and gating strategies.

☒ Tick this box to confirm that a figure exemplifying the gating strategy is provided in the Supplementary Information.

Magnetic resonance imaging

Experimental design

Design type

Indicate task or resting state; event-related or block design.

Design specifications

Specify the number of blocks, trials or experimental units per session and/or subject, and specify the length of each trial or block (if trials are blocked) and interval between trials.

Behavioral performance measures

State number and/or type of variables recorded (e.g. correct button press, response time) and what statistics were used to establish that the subjects were performing the task as expected (e.g. mean, range, and/or standard deviation across subjects).

Acquisition

Imaging type(s) — *Specify: functional, structural, diffusion, perfusion.*

Field strength — *Specify in Tesla*

Sequence & imaging parameters — *Specify the pulse sequence type (gradient echo, spin echo, etc.), imaging type (EPI, spiral, etc.), field of view, matrix size, slice thickness, orientation and TE/TR/flip angle.*

Area of acquisition — *State whether a whole brain scan was used OR define the area of acquisition, describing how the region was determined.*

Diffusion MRI ☐ Used ☐ Not used

Preprocessing

Preprocessing software — *Provide detail on software version and revision number and on specific parameters (model/functions, brain extraction, segmentation, smoothing kernel size, etc.).*

Normalization — *If data were normalized/standardized, describe the approach(es): specify linear or non-linear and define image types used for transformation OR indicate that data were not normalized and explain rationale for lack of normalization.*

Normalization template — *Describe the template used for normalization/transformation, specifying subject space or group standardized space (e.g. original Talairach, MNI305, ICBM152) OR indicate that the data were not normalized.*

Noise and artifact removal — *Describe your procedure(s) for artifact and structured noise removal, specifying motion parameters, tissue signals and physiological signals (heart rate, respiration).*

Volume censoring — *Define your software and/or method and criteria for volume censoring, and state the extent of such censoring.*

Statistical modeling & inference

Model type and settings — *Specify type (mass univariate, multivariate, RSA, predictive, etc.) and describe essential details of the model at the first and second levels (e.g. fixed, random or mixed effects; drift or auto-correlation).*

Effect(s) tested — *Define precise effect in terms of the task or stimulus conditions instead of psychological concepts and indicate whether ANOVA or factorial designs were used.*

Specify type of analysis: ☐ Whole brain ☐ ROI-based ☐ Both

Statistic type for inference — *Specify voxel-wise or cluster-wise and report all relevant parameters for cluster-wise methods.*

(See Eklund et al. 2016)

Correction — *Describe the type of correction and how it is obtained for multiple comparisons (e.g. FWE, FDR, permutation or Monte Carlo).*

Models & analysis

n/a	Involved in the study
☐	☐ Functional and/or effective connectivity
☐	☐ Graph analysis
☐	☐ Multivariate modeling or predictive analysis

Functional and/or effective connectivity — *Report the measures of dependence used and the model details (e.g. Pearson correlation, partial correlation, mutual information).*

Graph analysis — *Report the dependent variable and connectivity measure, specifying weighted graph or binarized graph, subject- or group-level, and the global and/or node summaries used (e.g. clustering coefficient, efficiency, etc.).*

Multivariate modeling and predictive analysis — *Specify independent variables, features extraction and dimension reduction, model, training and evaluation metrics.*
